# Pyridoxine Supplementation Improves the Activity of Recombinant Glutamate Decarboxylase and the Enzymatic Production of Gama-Aminobutyric Acid

**DOI:** 10.1371/journal.pone.0157466

**Published:** 2016-07-20

**Authors:** Yan Huang, Lingqia Su, Jing Wu

**Affiliations:** 1 State Key Laboratory of Food Science and Technology, Jiangnan University, Wuxi, China; 2 School of Biotechnology and Key Laboratory of Industrial Biotechnology Ministry of Education, Jiangnan University, Wuxi, China; Yeungnam University, REPUBLIC OF KOREA

## Abstract

Glutamate decarboxylase (GAD) catalyzes the irreversible decarboxylation of L-glutamate to the valuable food supplement γ-aminobutyric acid (GABA). In this study, GAD from *Escherichia coli* K12, a pyridoxal phosphate (PLP)-dependent enzyme, was overexpressed in *E*. *coli*. The GAD produced in media supplemented with 0.05 mM soluble vitamin B_6_ analog pyridoxine hydrochloride (GAD-V) activity was 154.8 U mL^-1^, 1.8-fold higher than that of GAD obtained without supplementation (GAD-C). Purified GAD-V exhibited increased activity (193.4 U mg^-1^, 1.5-fold higher than that of GAD-C), superior thermostability (2.8-fold greater than that of GAD-C), and higher *k*_cat_/*K*_m_ (1.6-fold higher than that of GAD-C). Under optimal conditions in reactions mixtures lacking added PLP, crude GAD-V converted 500 g L^-1^ monosodium glutamate (MSG) to GABA with a yield of 100%, and 750 g L^-1^ MSG with a yield of 88.7%. These results establish the utility of pyridoxine supplementation and lay the foundation for large-scale enzymatic production of GABA.

## Introduction

γ-Aminobutyric acid (GABA), a four carbon, non-essential amino acid that is widely distributed in nature, plays a major role as an inhibitory neurotransmitter in the mammalian central nervous system [1]. GABA has several physiological activities, including diuretic and tranquilizer effects, and has been used in the treatment of epilepsy and the prevention of obesity [2–5]. At present, GABA is widely used to reduce the concentration of the blood ammonia [1] and to treat hepatic coma [6]. GABA has also attracted interest for its potential use as a biopolymer precursor. For example, GABA can be converted to 2-pyrrolidone, a monomer of nylon 4 [7]. Due to its multiple functions, GABA is widely used in medicine, functional foods and the chemical industry.

The currently method of GABA production include enrichment from food, chemical synthesis and biosynthesis. The amount of GABA obtained by enrichment from food is quite low; it is used as a nutritional supplement in products such as GABA-green tea [8] and GABA-brown rice [9]. The chemical synthesis of GABA consumes large amounts of energy and is not environmental friendly [10]. The isolation of GABA-producing lactic acid bacteria from traditional fermented foods has attracted much interest recently. However, the highest level of GABA produced through fermentation has reached only 35.6 g L^-1^ [11–13]. Furthermore, the recovery of GABA from the complex fermentation broths required by these organisms is generally difficult and expensive to perform. These drawbacks have limited the widespread use of GABA.

In contrast, enzymatic synthesis, which uses the pyridoxal 5’-phosphate (PLP)-dependent enzyme glutamate decarboxylase (GAD) to catalyze the irreversible decarboxylation of L-glutamate to GABA, is performed under mild conditions with low cost and low energy consumption. Therefore, enzymatic synthesis is highly desirable as a method of industrial GABA production [14]. Due to the industrial importance of GABA, glutamate decarboxylases have recently been overexpressed in various hosts to improve GABA production. Among the GAD enzymes reported to date, the GAD from *E*. *coli* K12 exhibited the highest yield of GABA, reaching 280 g L^-1^ [15–20]. Therefore, *E*. *coli* GAD overexpressed in *E*. *coli* BL21 (DE3) was investigated further in the present study.

GAD requires PLP for its glutamate decarboxylase activity [21]. Although PLP can be generated by the phosphorylation of pyridoxal aldehyde in the bacterium, this route cannot meet the demands of recombinant GAD production. Therefore, most researchers add a certain amount of PLP to the enzymatic conversion mixture to assist in the production of GABA [20]. At present, only Plokhov et.al have reported a preparation method in which 0.02 mM PLP is added to the fermentation medium to enhance the yield of GAD. The production of GAD increased 2–2.5-fold in the presence of 0.02 mM PLP [19]. However, the high price, poor availability, and poor stability of PLP have limited its industrial application. A more suitable alternative is the vitamin B_6_ supplement pyridoxine hydrochloride (PN), a water soluble vitamin that exists widely in nature, can be taken up by cells, and is phosphorylated within the cell to form coenzyme PLP [22, 23]. In this study, a strain of *E*.*coli* BL21(DE3) harboring a GAD expression plasmid was constructed and used to overexpress *E*. *coli* GAD. The addition of PN to the fermentation medium significantly improved the production of GAD as well as its performance in GABA production. Thus, this process may, reduce the cost of GABA production, leading to vast commercial prospects.

## Materials and Methods

### Bacterial strains, plasmids, and materials

*E*. *coli* strains JM109 and BL21(DE3) were used as a cloning vehicle and an expression host, respectively. The EZ-10Spin Column Plasmid Mini-Prep kit and agarose gel DNA purification kit were purchased from Tiangen Co. Ltd (Beijing, China). The vectors pMD^™^18-T and pET-24a(+) were purchased from Novagen (Shanghai, China). The PrimeSTAR^®^HS DNA Polymerase, restriction enzymes and T4 DNA ligase were obtained from Takara (Dalian, China). Restriction endonuclease *Dpn*I was obtained from Generay Biotech (Shanghai, China). GABA, PLP, and 1,2-phthalic dicarboxaldehyde were obtained from Sigma Chemical Co. Ltd. (Shanghai, China). Other chemicals were purchased from Sinopharm Chemical Reagent Co. Ltd. (SCRC, Shanghai, China).

### Plasmid and strain construction

The *gadB* gene (NCBI accession number: NP_416010) of *E*. *coli str*. K12 *substr*. MG1655 was amplified from genomic DNA using a standard PCR method. The forward primer used during this process was 5’-CGCCATATGGACCAGAAGCTG TTAACGGAT-3’ (*Nde*I restriction site underlined), the reverse primer was 5’-CCGCTCGAGTCAGGTGTGTTTAAAGCTGTTCT-3’ (*Xho*I restriction site underlined). Amplification was performed with PrimeSTAR^®^HS DNA Polymerase. The amplification product was isolated and ligated directly into the cloning vector pMD18T-simple, which was then used to transform chemically competent *E*. *coli* JM109 cells. The identity of the plasmid was verified using enzyme restriction analysis, and its sequence was verified by DNA sequencing, which was conducted by Sangon Co. Ltd. The verified plasmid was digested with *Nde*I and *Xho*I, and the *gadB*-containing fragment was cloned into the expression vector pET-24a(+). The recombinant plasmid, *gadB*/pET-24a(+), was expanded in JM109 and used to transformed *E*. *coli* BL21(DE3) for overexpression.

### Expression of recombinant glutamate decarboxylase

*E*. *coli* BL21(DE3) containing the desired plasmid *gadB*/pET-24a(+) was grown in Luria-Bertani medium supplemented with 30 μg mL^-1^ kanamycin at 37°C for 8 h, with shaking at 200 rpm. This seed culture was diluted into the Terrific Broth medium and shaken at 200 rpm and 25°C. When the absorbance at 600 nm reached 1.5, enzyme production was induced by the addition of isopropyl-β-d-thiogalactopyranoside (IPTG) to a final concentration of 0.2 mM. The cells were incubated for an additional 24 h, during which the culture was sampled every 3 h. At the end of the 24 h incubation, the cells were collected by centrifugation at 12,000 g for 2 min at 4°C. These cells, which contain the enzyme produced in the absence of added PN (GAD-C) were used directly in the purification protocol described below.

To investigate the effect of PN on the functional properties of recombinant glutamate decarboxylase, the culture medium was supplemented with various concentrations of PN (final concentrations 0.01, 0.02, 0.03, 0.04, 0.05, and 1 mM) at the beginning of induction. Enzymes produced in the presence of PN (GAD-V) were also purified using the protocol described below.

### Purification of recombinant glutamate decarboxylase

The cell pellet was washed twice, then resuspended in 50 mM citric acid-Na_2_HPO_4_ buffer (pH 5.5), and then disrupted by ultrasonication (Vibra-cell; Sonics & Materials, Newtown, CT, USA) at 20 kHz for 10 min in an ice bath. The lysate was clarified by centrifugation at 10,000 g for 10 min at 4°C. Solid ammonium sulfate was slowly added into the supernatant, with stirring, to a final concentration of 60% (w/v). The solution was kept at 4°C overnight to precipitate proteins. The precipitate was collected by centrifugation and dissolved in buffer A (20 mM citric acid-Na_2_HPO_4_ buffer, pH 6.5, containing 0.15 mM PLP) and dialyzed against 1 L of buffer A at 4°C overnight. The sample was filtered (0.22 μm) and loaded onto a DEAE-Sepharose column pre-equilibrated with buffer A. The column was eluted using a linear gradient from 0–1 M NaCl in buffer A at the flow rate of 1.0 mL min^-1^. The fractions containing glutamate decarboxylase activity were pooled and dialyzed against 1 L of buffer A at 4°C.

The homogeneity and subunit molecular weight of the purified protein were assessed by denaturing SDS-PAGE. Protein bands were visualized by staining with 0.25% Coomassie Brilliant Blue R-250. Densitometry was performed by scanning the gel and analyzing the scanned image using Quantity One 4.6.2 software (Bio-Rad Laboratories, Calif, USA) gel. Protein concentration was determined using the Bio-Rad protein assay kit (Bio-Rad), with purified bovine serum albumin (Promega) as the standard. The purified enzyme was stored at −20°C.

### Glutamate decarboxylase activity assay

Glutamate decarboxylase activity was determined by measuring the amount of GABA produced, using HPLC. One unit (U) of glutamate decarboxylase activity was defined as the amount of enzyme that liberates 1μmol GABA per minute under the following activity assay condition. The reaction mixture contained, in a final volume of 400 μL, 100 mM glutamic acid, 50 mM Citric-Na_2_HPO_4_ buffer (pH 4.8), and 0.15 mM PLP. Reaction was initiated by addition of an aliquot of the enzyme. After being incubated at 37°C for 4 min, reaction was stopped by adding 600 μL of 0.2 M borate, pH 10.0, and boiling for 10 min to inactivate the enzyme. The supernatant obtained by centrifugation of the reaction mixture at 12,000 g for 10 min at room temperature was analyzed using the HPLC assay procedure described in section HPLC assay of GABA. The glutamate decarboxylase activity reported represent the means of three independent measurements ± their standard deviations.

### pH optimum and stability

The effect of pH on glutamate decarboxylase activity was measured between pH 3 and pH 7 using the assay method described in section glutamate decarboxylase activity assay, except that the reaction buffer, 50 mM citric acid-Na_2_HPO_4_, was adjusted to pH values between pH 3.0 and pH 7.0 in increments of 1 pH unit. To determine the stability of the enzyme at these pH values, samples were incubated at 4°C in each of the reaction buffers described above, supplemented with 0.15 mM PLP for 24 h. At the end of the incubation period, the residual GAD activity in each sample was assessed using the assay described in section glutamate decarboxylase activity assay.

### Temperature optimum and thermostability

The effect of temperatures on enzyme activity was determined using the assay described in section glutamate decarboxylase activity assay, except at temperatures ranging from 30°C to 60°C. Prior to initiation of the reaction, the substrate solution and the enzyme were pre-incubated separately at the appropriate temperature for 7 min. The reaction was initiated by the addition of the enzyme and allowed to proceed for 4 min.

The thermostability of an enzyme was determined by incubating the enzyme in 50 mM citric acid-Na_2_HPO_4_ buffer, pH 4.8, containing 0.15 mM PLP, at 37°C. The reaction mixture was sampled at regular intervals and assayed for residual activity using the method described in section HPLC assay of GABA.

### Kinetic studies

Reaction kinetics for GAD-C and GAD-V were determined using the glutamate decarboxylase assay described in section glutamate decarboxylase activity assay, except that the glutamic acid concentration typically ranged from 5 mM to 200 mM. The rate versus substrate concentration data were fit to the Michaelis-Menten equation by nonlinear regression using GraphPad Prism version5.0 software. The *K*_m_ and *k*_cat_ values reported represent the means of three independent measurements ± their standard deviations.

### Size exclusion chromatography

Size exclusion chromatography was conducted using an AKTA avant 25 System (GE Healthcare, USA) equipped with a Superdex 200 10/300GL column (GE Healthcare, USA). The elution buffer was 20 mM citric acid-Na_2_HPO_4_ buffer (pH 6.5, containing 0.15 mM PLP). The purified GAD-C and GAD-V were eluted at a flow rate of 0.4 mL/min and the column effluent was monitored using UV spectrometry at 280 nm. Five peptides, including Blue Dextran, Apoferritin from horse spleen, β-amylase, Alcohol Dehydrogenase from yeast and Carbonic Anhydrase from bovine were used as molecular weight standards (Sigma-Aldrich, Shanghai, China).

### Circular Dichroism Analysis

Circular dichroism (CD) analysis was carried out with a BioLogic Mos-450 spectropolarimeter (BioLogic Science Instrument, Grenoble, France) equipped with a 1 mm quartz cuvette. The samples were diluted to a protein concentration of 0.05–0.20 mg/mL in 20 mM citric acid-Na_2_HPO_4_ buffer (pH 6.5, containing 0.15 mM PLP). Ellipticity data of the enzymes was continuously collected in the far-ultraviolet (195–250 nm) spectrum. Secondary structure content was calculated from the CD spectrum using the CDSSTR algorithm on DichroWeb (http://www.cryst.bbk.ac.uk/cdweb)[24].

### Optimization of the reaction conditions for GABA production

In order to optimize the enzymatic production of GABA from L-glutamate, a series of reactions confirming the optimal pH, temperature, enzyme concentration and substrate concentration were carried out. Monosodium glutamate (MSG), which has higher solubility than that of glutamic acid, was chosen as the substrate. MSG (250 g L^-1^) was dissolved in 50 mM citric acid-Na_2_HPO_4_ buffer, at the pH indicated, containing 0.15 mM PLP to a final volume of 10 mL. The reaction was carried out in a constant temperature incubator shaker with continuous agitation at 150 rpm. The reaction was terminated by the addition of an equal volume of 1 mol·L^-1^ NaOH. The reaction products were then separated and quantified by HPLC, as described in section HPLC assay of GABA. All data are the average of three independent experiments.

### GABA synthesis with crude enzyme GAD

The synthesis of GABA with crude enzyme, which contained an unknown concentration of PLP, was tested in a 1 L reaction system. Under optimal conditions of section optimization of the reaction conditions for GABA production, MSG (250 g L^-1^) dissolved in 50 mM citric acid-Na_2_HPO_4_ buffer, pH 5.0, was used as initial concentration. After the first 4 h, an additional 50 g L^-1^ MSG was added every 2 h until the total amount of MSG added reached 500 g L^-1^ or 750 g L^-1^. After the final substrate addition (at 500 and 750 g L^-1^, respectively), the reaction was continued for additional 12 hours. The reaction was terminated by the addition of an equal volume of 1 mol·L^-1^ NaOH. The final concentrations of MSG and GABA were determined using the HPLC protocol described in section HPLC assay of GABA. All data are the average of three independent experiments.

### HPLC assay of GABA

The amounts of GABA and glutamic acid or MSG present in reaction mixtures were determined using HPLC with an Agilent 1200 HPLC system (Agilent Technologies, Palo Alto, CA, USA) equipped with an Eclipse XDB-C18 5 μm (4.6 mm × 150 mm) column (Agilent Technologies). The analyses were conducted with gradient elution using a mobile phase formed with buffer A (4.52 g L^-1^ anhydrous sodium acetate, 200 μL L^-1^ triethylamine, 5 mL L^-1^ tetrahydrofuran, pH 7.2 ± 0.05) and buffer B (22.6 g L^-1^ sodium acetate anhydrous, pH 7.2 ± 0.05/acetonitrile/methanol = 1:2:2, by volume), described by Chen et al. [25]. The column temperature was maintained at 40°C, the flow rate was 0.8 mL min^-1^ and the products were detected at 338 nm with a UV monitor. Samples containing 1 mM GABA (Sigma) and 1 mM glutamic acid (SCRC, Shanghai, China) were used as standards. All data are the average of three independent experiments.

## Results and Discussion

### Cloning and expression of GAD in *E*.*coli* BL21(DE3)

**Fig 1:**
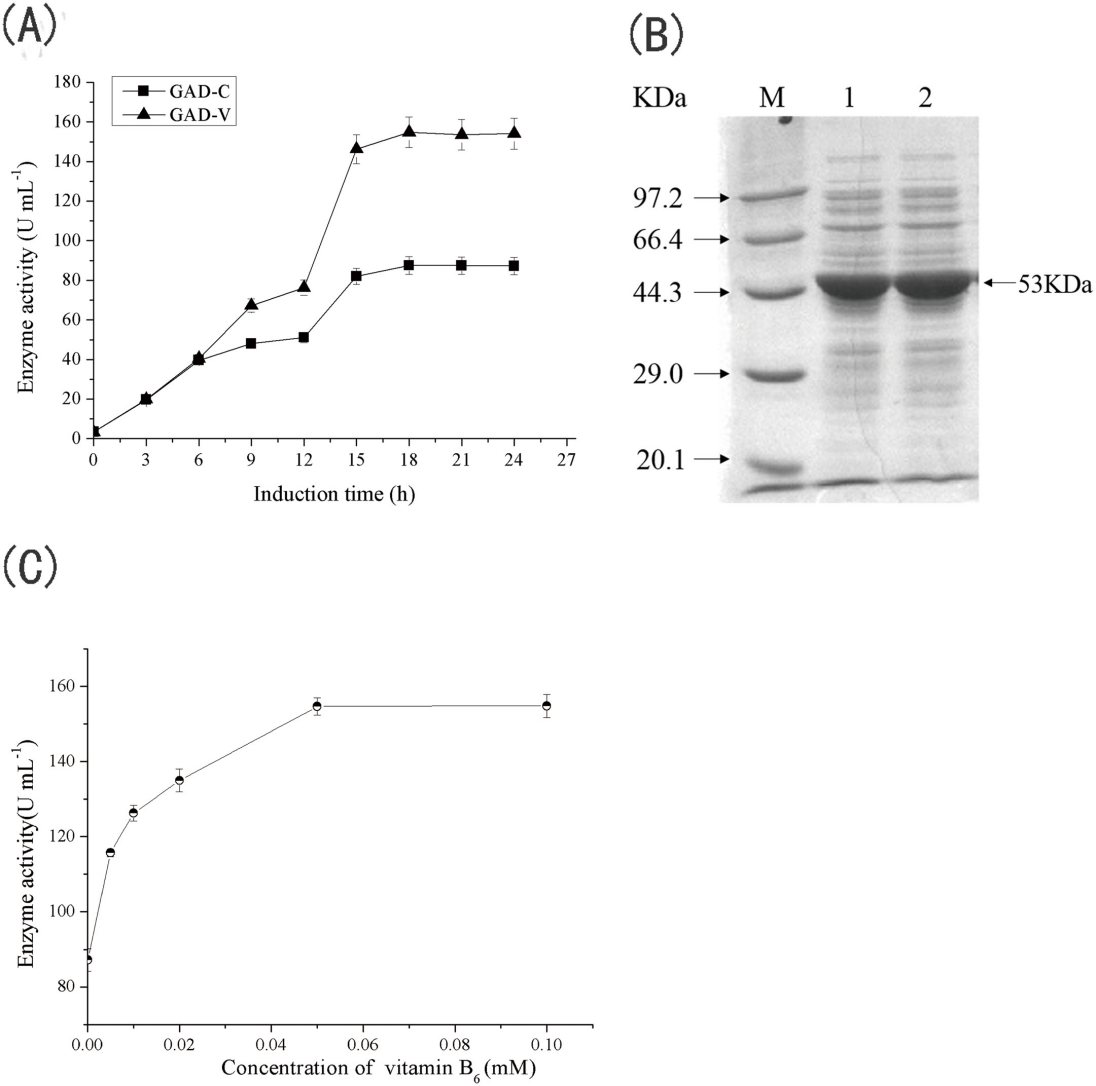
Effects of PN on the production of GAD. (A) Effects of PN on the activity of GAD. (B) SDS-PAGE analysis of GAD expression in *E*.*coli*. Lane M, molecular weight markers; lane1, Intracellular soluble fraction from culture in TB medium; lane 2, Intracellular soluble fraction from culture in TB medium supplemented with PN. (C) Effects of different concentration of pyridoxine hydrochloride on GAD production. Error bars represent the standard deviations from three independent measurements. The figure was generated using Origin 9.0.

The gadB gene (NCBI accession number: NP_416010) was amplified from the chromosomal DNA of *E*.*coli* str.K12 *substr*.MG1655 using PCR, and inserted into the expression vector pET-24a(+). The resulting recombinant plasmid, gadB/pET-24a, was used to transform *E*. *coli* BL21(DE3) for GAD expression. When this recombinant *E*. *coli* was cultivated in standard TB medium and GAD expression was induced with IPTG, the GAD activity in the cytoplasm increased gradually, reaching its highest value (87.5 U mL^-1^) 18 h after induction (Fig 1A). SDS-PAGE analysis the cytoplasmic fraction showed a major band at approximately 54 kDa (Fig 1B), which is consistent with the molecular weight of GAD previously reported [20, 26] This enzyme, which was used as the control for further experiments, will be referred to as GAD-C.

### Effect of PN on the production of GAD

In an initial experiment, the above-mentioned recombinant *E*. *coli* was cultured in TB medium that was supplemented at the beginning of induction with PN at final concentrations of 0.005–0.1 mM. Enzymes produced in the presence of PN will be referred to as GAD-V, to distinguish them from GAD-C. As shown in Fig 1C, when the concentration of PN was below 0.05 mM, the GAD-V activity tended to increase as the PN concentration increased. The highest activity (154.8 U mL^-1^), which is 1.8-fold higher than that of GAD-C, was obtained when the culture medium was supplemented with 0.05 mM PN. Further analysis of the fermentation process showed that cells grown under these two conditions exhibited essentially the same growth rate. The activities of GAD-C and GAD-V showed similar profiles for the first 8 h post-induction (Fig 1A), but the GAD-V activity increased to a greater extent than the GAD-C activity thereafter. These results suggest that the natural intracellular PLP concentration is sufficient for incorporation into the GAD being rapidly expressed from the T7 promoter system during the early stages of expression. However, as induction proceeded, the elevated GAD expression level required an accordingly increased amount of PLP, which could not be met without exogenous addition of PLP or a PLP precursor. Thus, the addition of PN to the medium enhanced the activity of GAD-V.

Although GAD activity was enhanced by the addition of PN, the protein bands corresponding to GAD-V and GAD-C in the SDS-PAGE analysis were similar in both size and intensity (Fig 1B). The total protein concentrations found in the cell lysates were 0.87 mg mL^-1^ and 0.93 mg mL^-1^ for GAD-C and GAD-V, respectively. The proportions of each protein within the crude lysate, analyzed using densitometric analysis of the SDS-PAGE gel, were 90.2% and 91.5% for GAD-C and GAD-V, respectively. Thus, detailed analysis of the crude lysate demonstrated that the addition of PN created no obvious difference in protein concentration of expression. Therefore, the increased enzyme activity displayed by GAD-V must be the result of enhanced specific activity that resulted from the addition of PN.

### Purification of GAD

**Fig 2:**
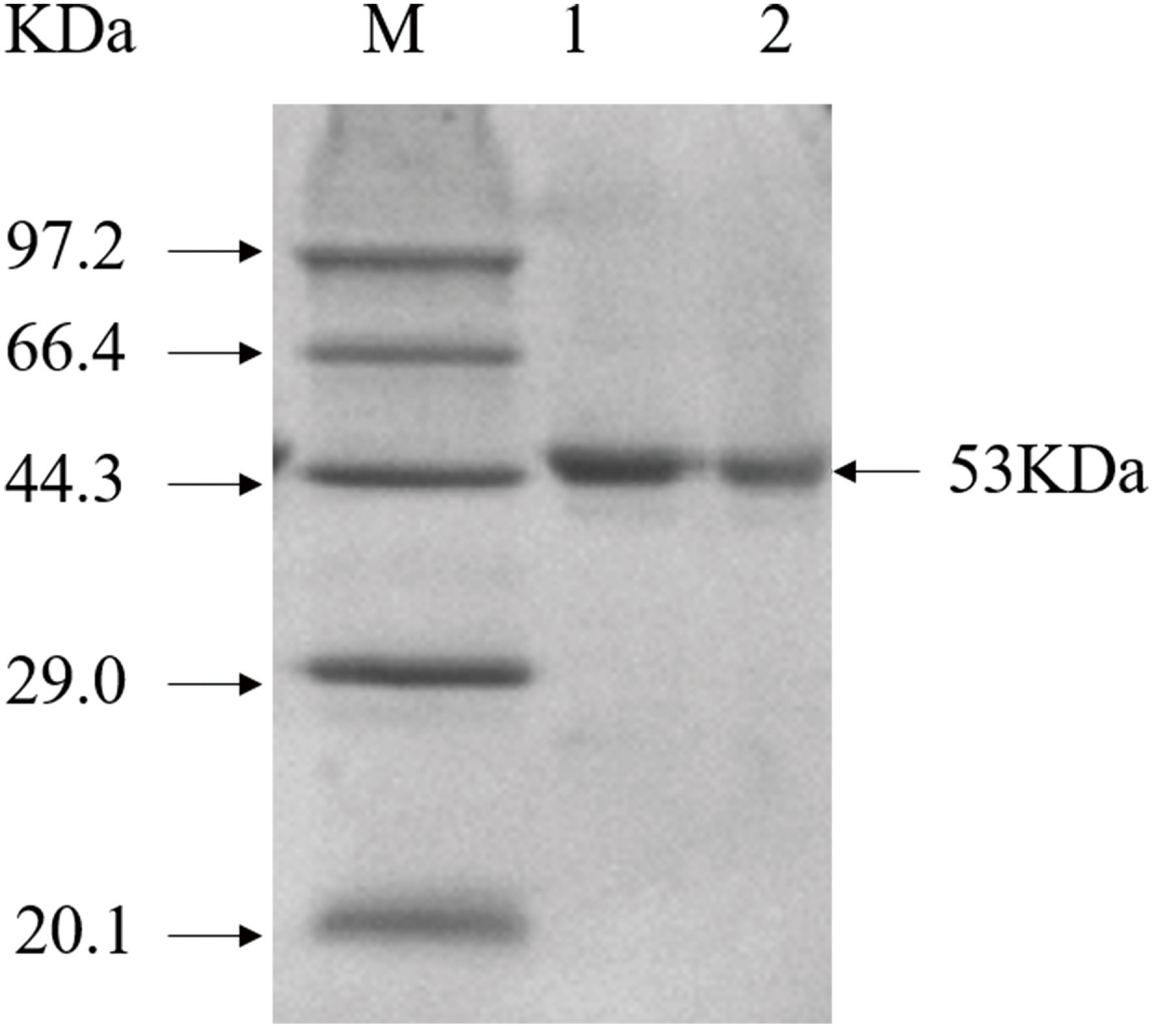
The purification of GAD. Lane M, molecular weight marker; lane 1, GAD-C; lane 2, GAD-V.

GAD-C and GAD-V were purified by ammonium sulfate precipitation and DEAE-Sepharose anion exchange chromatography (Table 1). The purified enzymes appeared to be homogeneous by SDS-PAGE (Fig 2). The specific activity of GAD-V was determined to be 193.4 U mg^-1^, which was 1.5-fold higher than that of GAD-C (130.2 U mg^-1^). This is consistent with our hypothesis that the addition of PN to the medium would increase the specific activity of the GAD produced. It has been reported that the GAD is depended on the cofactor of PLP for its activity. PLP forms a covalent bond with the ɛ-amino group of a conserved lysine residue at the active site of GAD [26]. Supplementation of the cytosol with sufficient PLP, accomplished by supplementing the medium with PN, is likely to have increased the proportion of GAD molecules containing bound PLP during protein folding. This would result in an increase in properly folded GAD molecules, and therefore higher specific activity. Further studies are underway to clarify this issue.

**Table 1:** Summary of the purification of GAD.

(a) Purification of recombinant GAD-C					
Purification step	Total Protein (mg)	Total activity (U)	Activity recovery (%)	Specific activity (U mg^-1^)	Purification (fold)
Crude extract	70.2	5307.1	100	75.2	1
Ammonium sulfate	30.2	3020.1	57	100.4	1.3
DEAE Sepharose Anion exchange	7.5	989.6	18.7	130.2	1.7
(b) Purification of recombinant GAD-V					
Purification step	Total protein (mg)	Total activity (U)	Activity recovery (%)	Specific activity (U mg^-1^)	Purification (fold)
Crude extract	67.6	6983.1	100	103.3	1
Ammonium sulfate	26.3	3779.3	53.4	143.7	1.4
DEAE Sepharose anion exchange	6.9	1334.5	19.0	193.4	1.9

### Potential mechanism of the increased activity of GAD-V

The above results showed that the activity of the GAD-V produced in media supplemented with 0.05 mM PN was 1.8-fold higher than that of GAD-C obtained without supplementation. One possible reason was that the PN has active effects on the enzyme. In our study, we added PN to the purified GAD-C and GAD-V in vitro, and analyzed the enzymatic activity. Results showed no difference of the activities of GADs with addition of PN or not. The potential hypothesis for the different activity of GAD-C and GAD-V probably caused by some change in the structure of the enzyme.

**Fig 3:**
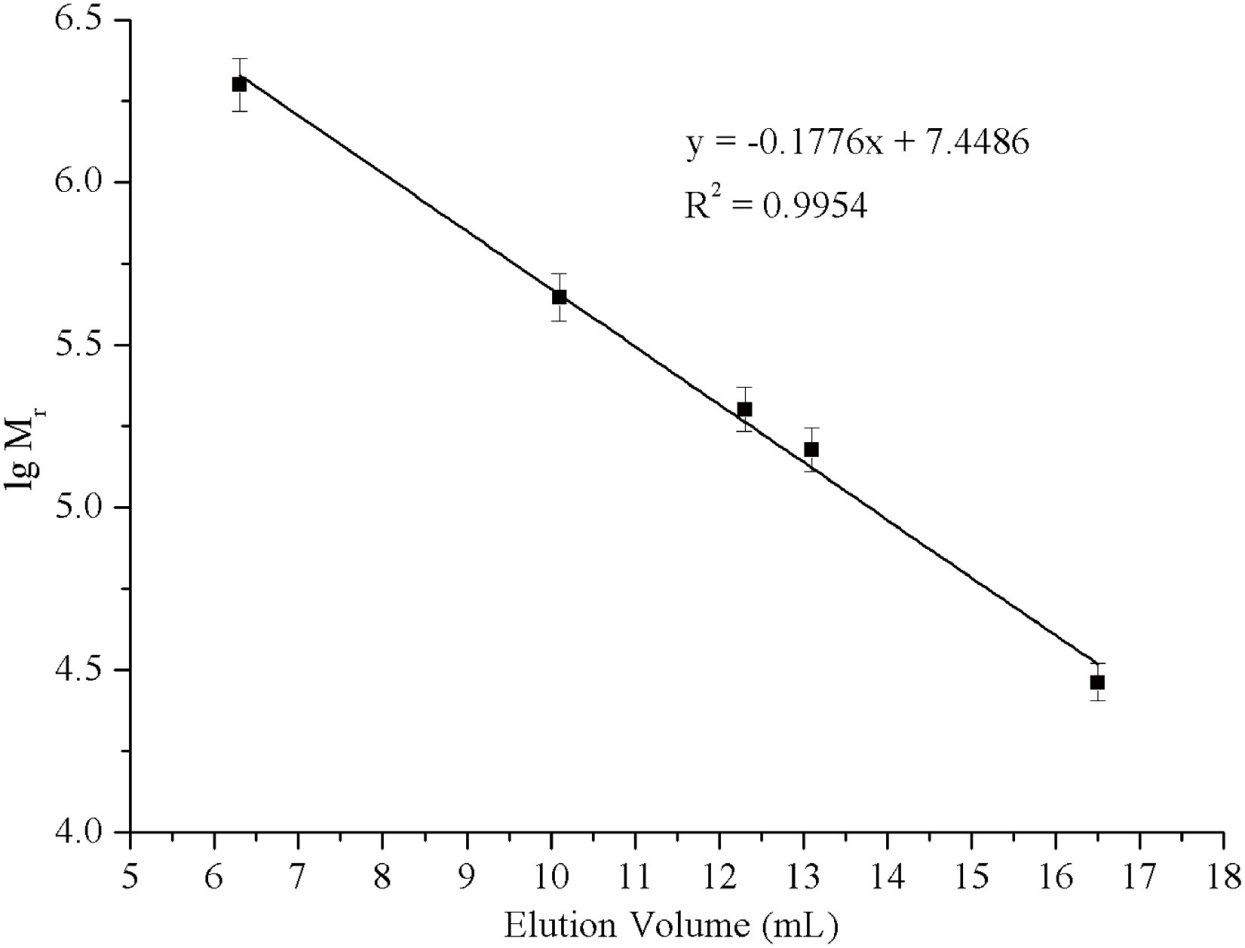
Gel filtration chromatogram of enzyme. Calibration curve of molecular weight versus elution volume. Standard: 1, Blue Dextran; 2, Apoferritin from horse spleen; 3, β-amylase; 4, Alcohol Dehydrogenase from yeast; 5, Carbonic Anhydrase from bovine.

**Fig 4:**
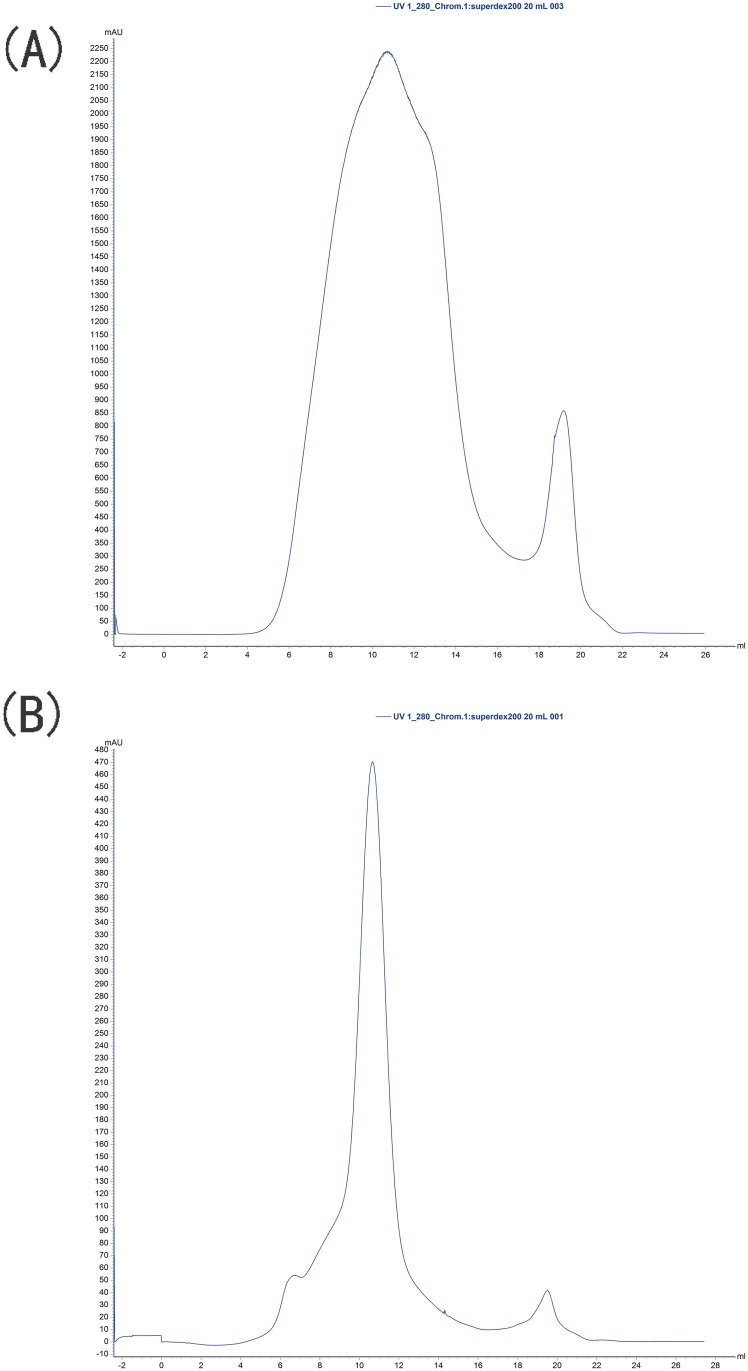
Gel filtration chromatograms of GAD-C(A) and GAD-V(B). The blue line represents the absorbance peak at 280 nm. Both the elution volumes of GAD were 11.2 mL.

The formation of aggregates of GAD-C and GAD-V was analyzed by Size exclusion chromatography. The calibration curve (Fig 3) of molecular weight versus elution volume was obtained with the molecular weight standards. As shown in Fig 4, the native molecular weight of the GAD-V and GAD-C protein were nearly same, were about 324 kDa, both GAD proteins form hexamers in solution, which is consistent with the molecular weight of GAD previously reported [27]. These results indicate that the addition of PN did not influence the aggregation state of GAD.

**Fig 5:**
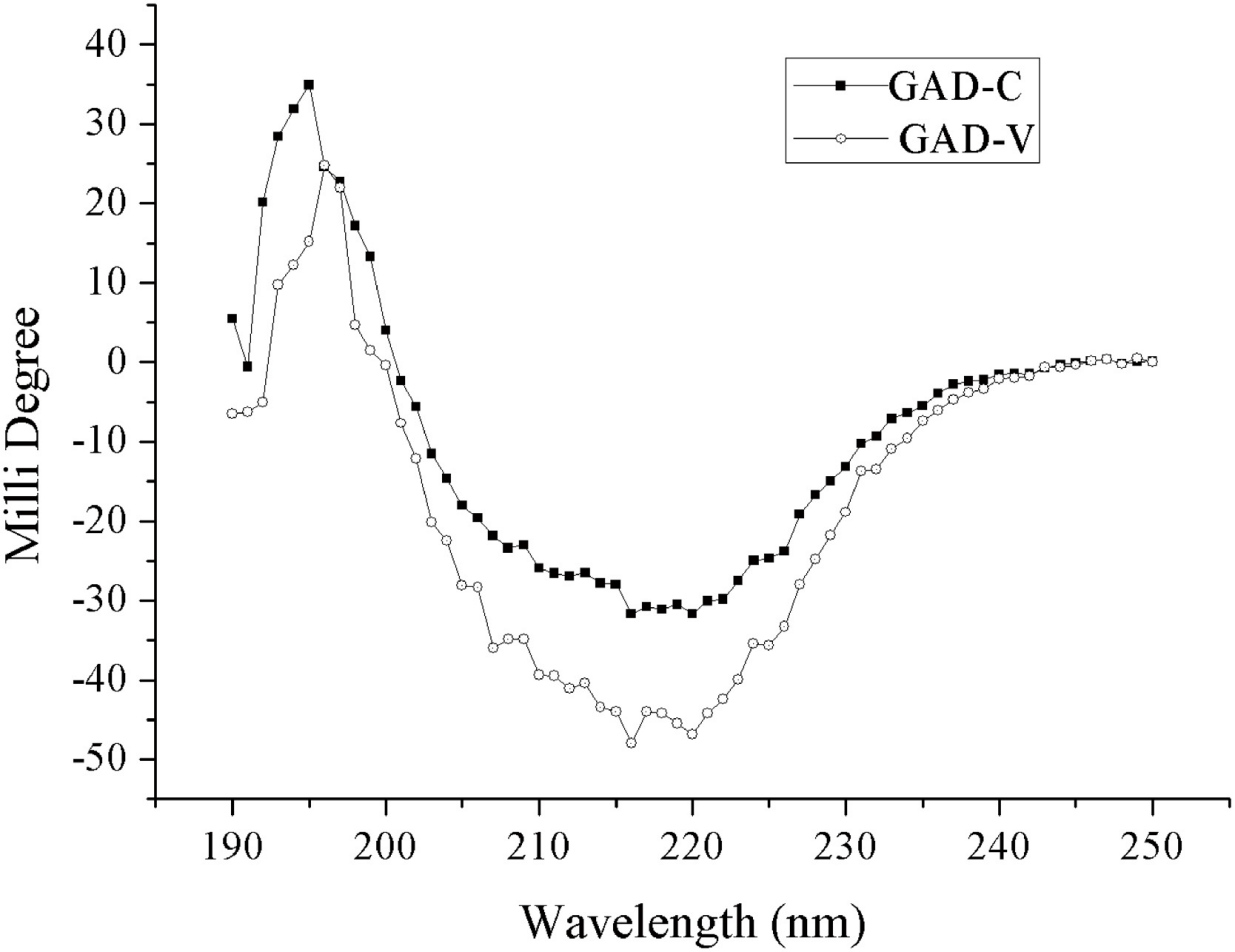
CD spectra of GAD in 20 mM citric acid-Na_2_HPO_4_ buffer (pH 6.5, containing 0.15 mM PLP).

The secondary structure of the GADs was investigated by Circular Dichroism Analysis (CD), and results showed that GAD-V contains a higher content of α-helix (54% versus 47%) and a lower content of β-sheet (20% versus 27%) than GAD-C (Fig 5 and Table 2). *E*. *coli* GAD is a typical of PLP-dependent enzyme. The PLP binding site exhibits an α/β fold consisting of a central seven-stranded mixed β-sheet surrounded by eight α-helices[28]. The aldehyde group of PLP could be linked with the epsilon-amino group of the active site lysine residue of the GAD, which is critical to the enzymatic activity[21]. Previous reports showed that α-helix helps to stabilize the binding of PLP[29]. As for the GAD-V, the increase of α-helix may occur in or near the PLP binding site, so the GAD-V could combine more easily and stably with PLP, which led to a higher specific activity of GAD-V. More experiments are underway to further explore the mechanism.

**Table 2:** Content of secondary structures predicted by CD.

Proteins	Helix (%)	Strand (%)	Turns (%)	Disordered (%)
GAD-C	47.0	27.0	6.0	20.0
GAD-V	54.0	20.0	6.0	20.0

### Enzymatic properties of GAD

#### pH optimum and pH stability

**Fig 6:**
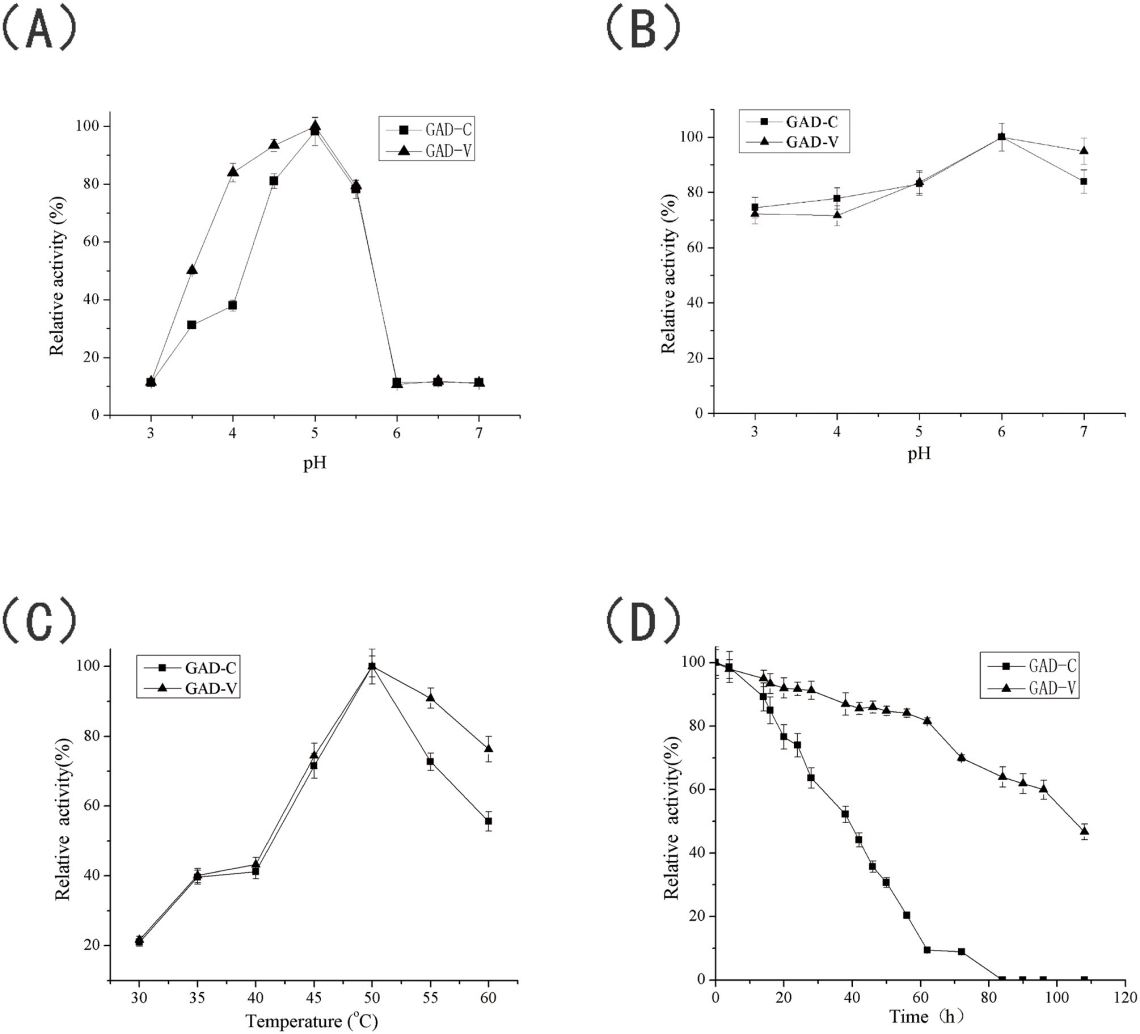
Comparison of enzymatic properties. (A) pH optimum. The activities of GAD-C and GAD-V were determined in 50 mM citric acid-Na_2_HPO_4_ buffer (pH 3.0–7.0). The activity at optimal pH was taken as 100%. (B) pH stability. Samples were assayed after incubation for 24 h at 4°C in 50 mM citric acid-Na_2_HPO_4_ buffer (pH 3.0–7.0). The activity without preincubation was taken as 100%. (C) Optimum temperature. The activities of the GAD-C and GAD-V were determined at 30–60°C. The activity at optimal temperature was taken as 100%. (D) Thermostability. Both of the GAD-C and GAD-V were incubated at 37°C in 50 mM citric acid-Na_2_HPO_4_ buffer (pH 4.8). The activity without preincubation was taken as 100%. Error bars correspond to the standard deviation of three independent determinations. The figure was generated using Origin 9.0.

The effect of pH on GAD activity was investigated from pH 3.0 to pH 7.0 (Fig 6A). Purified GAD-C and GAD-V showed very similar responses to changes in pH. They both showed optimal activity at pH 5.0, and their activities decreased dramatically between pH 5.0 and pH 7.0. GAD-V exhibited a much higher activity than GAD-C at pH below 5.0. More than 80% of the maximum catalytic activity was observed for GAD-V between pH 4.0 and 5.0, while GAD-C exhibited 98.3% and 38% of the activity shown by GAD-V at pH 5.0 and 4.0, respectively. According to previous reports, a pH range of 4.0–5.0 is generally appropriate for the preparation of GABA [11, 13, 14, 20, 30]. Thus, the higher activity of GAD-V under these conditions make GAD-V more suitable for the large-scale preparation of GABA.

The pH stabilities of the recombinant enzymes were measured by incubating them in 50 mM citric acid-Na_2_HPO_4_ buffers between pH 3.0 and 7.0 at 4°C for 24 h. Both GAD-C and GAD-V retained more than 70% of their maximal activity between pH 3.0 and 5.0 and more than 80% between pH 5.0 and 7.0 (Fig 6B).

#### Temperature optimum and thermostability of GAD

The optimal temperatures for purified GAD-C and GAD-V were determined by measuring the enzyme activities in temperatures between 30 and 60°C. Both GAD-C and GAD-V exhibited an optimal temperature of 50°C, and the relative activities of GAD-C and GAD-V were almost the same below 50°C. When the temperature was higher than 50°C, the activity of GAD-V was almost 20% higher than that of the GAD-C (Fig 6C).

The thermostabilities of GAD-C and GAD-V were determined by incubating the enzymes at 37°C and measuring their residual activities at different time intervals. As shown in Fig 6D, the activity of GAD-C decreased rapidly with time, with a half-life of 38 h. In contrast, GAD-V showed a half-life of 108 h, which was about 3-fold that of GAD-C. As noted above, supplementation of cellular PLP is expected to increase the proportion of GAD molecules modified with this critical cofactor. Molecules lacking the cofactor are expected to fold improperly and may, therefore, be more labile upon heating. The presence of a substantial amount of unfolded protein may accelerate the loss of GAD-C activity through non-specific aggregation. The improved thermostability of GAD-V is an advantage in the storage and application of the enzyme. To the best of our knowledge, this is the first report on the improvement of GAD thermostability through addition of PN.

#### Kinetic studies

The kinetic parameters of the purified enzymes were analyzed using glutamic acid as the substrate at 37°C. The *K*_m_ and *k*_cat_ values of GAD-C were 14.8 mM and 102.2 s^−1^, respectively, which are similar to those reported by Ngoc et al. (12.8 mM and 94.6 s^−1^, respectively) [31]. The *K*_m_ values of GAD-V (16.3 mM) showed slight difference with that of GAD-C, but the *k*_cat_ of GAD-V (183.3 s^−1^) was 1.8-fold higher than that of the GAD-C. Correspondingly with that, the *k*_cat_/*K*_m_ value of GAD-V (11.2 mM^−1^s^−1^) was 1.6-fold of that of GAD-C (6.9 mM^−1^ s^−1^). This difference is likely related to an increased proportion of PLP-containing molecules within GAD-V, compared with GAD-C.

### Optimization of the reaction conditions for GABA production by the purified GAD

Because our ultimate aim is to improve the large-scale production of GABA from MSG, the reaction conditions for the conversion of MSG to GABA using elevated MSG concentrations were optimized. In an effort to enhance the activity this PLP-dependent enzyme, 0.15 mM PLP was added to the reaction mixtures.

#### Effect of pH on GABA production

**Fig 7:**
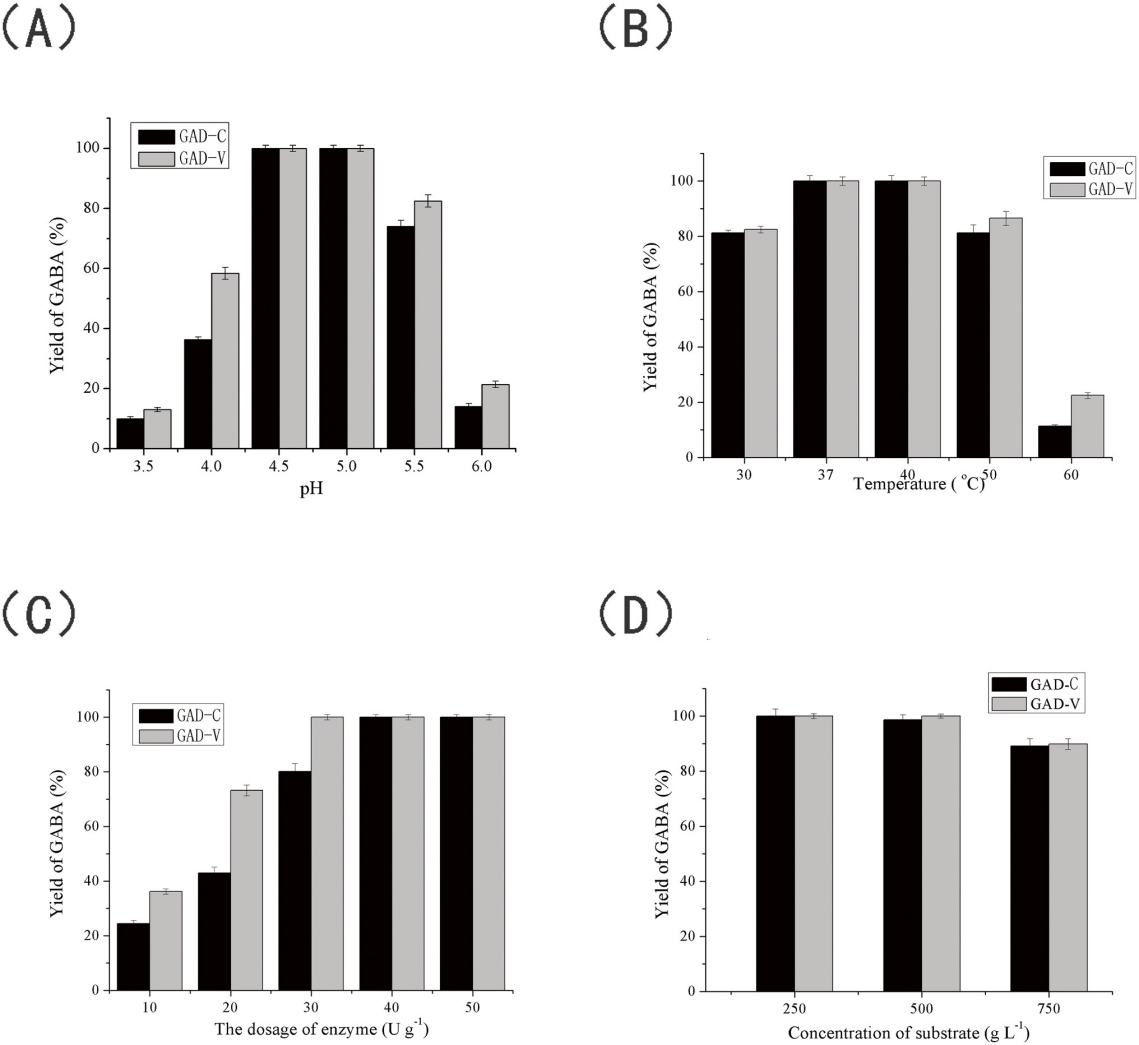
Optimization of the reaction conditions for GABA production using purified GAD. (A) Effect of pH on GABA production. (B) Effect of temperature on GABA production. (C) Effect of enzyme dosage on GABA production. (D) Effect of substrate concentration on GABA production. Error bars represent the standard deviations from three independent determinations. The figure was generated using Origin 9.0.

The optimal pH for the biotransformation of MSG to GABA was investigated between pH 3.5 and 6.0. As shown in Fig 7A, the yields of GABA reached 100% at pH 4.8 and 5.0 for both the GAD-C and GAD-V. The yields gradually decreased at pH lower than 4.8 and higher than 5.0, probably due to reduced catalytic activity. However, at the concentration used in these experiments (250 g L^-1^) the MSG substrate does not completely dissolve at pH lower than 4.8; it tends to crystallize, which was not conducive for the reaction. Therefore, subsequent reactions were conducted at pH 5.0.

#### Effect of temperature on GABA production

The temperature optimum of GABA production was studied at temperatures ranging from 30 to 60°C. As shown in Fig 7B, both GAD-C and GAD-V gave 100% yield at 37°C and 40°C, and more than 80% between 30°C and 50°C. However, the yields decreased dramatically, to less than 20%, at 60°C. This decrease probably resulted from enzyme inactivation at high temperature. Subsequent experiments were performed at 37°C to maintain consistency with other reports [18–20].

#### Effect of enzyme dosage on GABA production

The optimal enzyme dosage for GABA production was studied from 10 to 50 units per gram of substrate (U g^-1^). As shown in Fig 7C, the yields of GABA in reactions catalyzed by GAD-C and GAD-V increased with the enzyme dosage, and reached 100% at 40 U g^-1^and 30 U g^-1^, respectively. After that, increased enzyme dosage only shortened the conversion time. In order to minimize preparation costs, dosages of 40 U g^-1^ and 30 U g^-1^ were chosen as optimal for GAD-C and GAD-V, respectively.

#### Effect of substrate concentration on GABA production

In order to enhance the productivity of GABA, the effect of the increased substrate concentration of 250 to 750 g L^-1^ was investigated. Preliminary experiments showed that the solubility of MSG was 250 g L^-1^ at pH 5.0. Attempts to dissolve greater amounts led to the formation of a white emulsion, which drastically decreased the yield of GABA (15%). This decrease was may have been due to the protein may bind to the crystals and become inactivated as well. To avoid this precipitation issue, a fed batch method was applied to GABA production. In this method, 250 g L^-1^ MSG was used as the initial substrate concentration and the reaction was allowed to proceed for 4 h. At this point, 50 g L^-1^ MSG was added into the reaction system every 2 h to reach a final substrate concentration of either 500 g L^-1^ or 750 g L^-1^, and then the reaction was allowed to proceed for an additional 12 h. As shown in Fig 7D, when the concentration of MSG was 500 g L^-1^ and 750 g L^-1^, the yields of GABA for GAD-V were 100% and 89.9%, respectively, while it was 97.2% and 89.2% for GAD-C, respectively. These results indicate that the purified GAD samples showed comparable GABA synthesis (100% yield) with a substrate concentration of 500 g L^-1^, under their respective optimal reaction conditions, when an adequate concentration of PLP is supplied in the reaction. When the substrate concentration was raised 750 g L^-1^, the enzymes still exhibited substantial yield (89.2% and 89.9%, respectively). The yield of GABA reached 378 g L^-1^ under optimal conditions, which is the highest ever reported [19].

### GABA production by the crude extract of GAD

The above results showed that GABA could be efficiently produced by recombinant GAD in the presence of adequate PLP, but that this approach is not practical for large-scale applications due to the high-cost of PLP. Therefore, the production of GABA by the crude extracts, GAD-C and GAD-V, which contain unknown amounts of PLP, was investigated using different concentrations of MSG under the optimized conditions.

**Fig 8:**
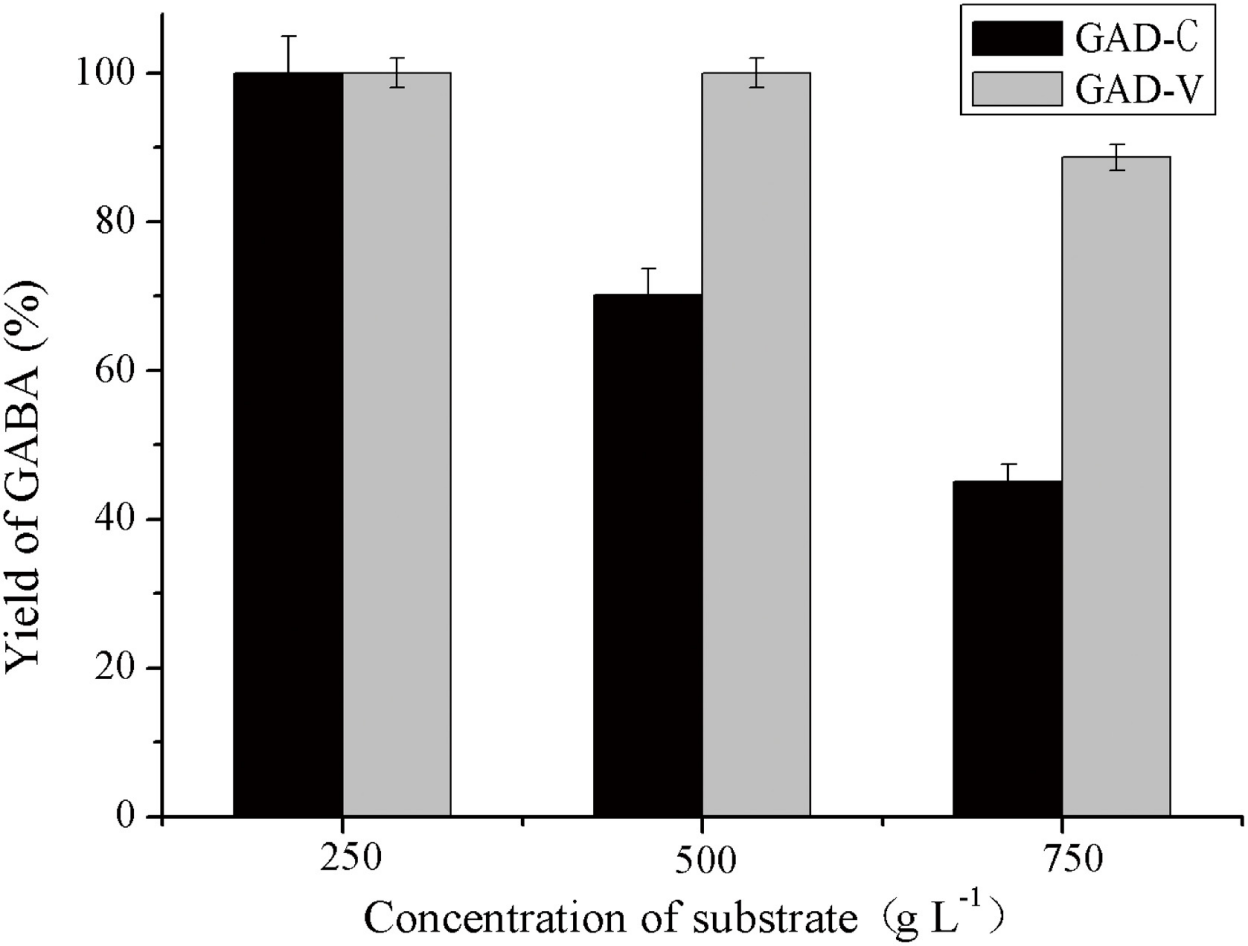
GABA synthesis with crude enzyme GAD. Error bars represent the standard deviations from three independent determinations.

As shown in Fig 8, at a substrate concentration of 250 g L^-1^, the yield of GABA for both crude GAD-C and GAD-V was 100%. But as the substrate concentration increased, the yield of GABA decreased significantly to 70.2% and 45.1% for GAD-C at the substrate concentration of 500 g L^-1^ and 750 g L^-1^, respectively, while it was 100% and 88.7% for GAD-V, respectively, which was very close to that for the purified GAD-V with 0.15 mM PLP.

As above mentioned, when PN is added in the culture medium, it is absorbed by the cells and phosphorylated to PLP, which is necessary for the catalytic activity of GAD. In addition, the activity of crude GAD-C and GAD-V were determined without additive PLP in the reaction mixture, compared with glutamate decarboxylase assay described in section glutamate decarboxylase activity assay. It was found that the activity of the crude extract of GAD-C and GAD-V without additive PLP was 30% and 90% of that with adequate PLP, respectively. Because there PLP was not added to the enzymatic synthesis reaction, the actual activity of GAD-C was smaller than that of GAD-V, is consistent with the optimal enzyme dosages. In addition, the thermostability of GAD-V in the crude extract was much higher than that of the GAD-C, with half-lives of 70 and 21 h, respectively. The thermostability decreased in the hyperosmotic environment caused by the high concentration of reactants, such as the 750 g L^-1^ glutamate (data not shown). Except for the improved thermal stability of GAD-V, which was probably due to the improved protein folding state, the much higher concentration of PLP contained in the crude extract of GAD-V may also benefit the GAD stability. The combined effects enhance GABA production by the crude extract of GAD-V.

In this research, the widely available and inexpensive vitamin B_6_ supplement PN was added during the fermentation process to obtain crude GAD that, without even trivial purification procedures, could be used directly for production of GABA. This approach not only simplified the process of enzymatic conversion, but also overcame the disadvantages caused by limited source, high cost and instability of PLP in practical use, which lays the foundation for the large-scale production of GABA.

## Conclusion

In this study, we investigated the effects of PN on the production, characteristics and application of GAD. We found that the addition of PN improved the specific activity by 1.5-fold and enhanced the thermostability by 2.8-fold, compared with that of the enzyme prepared without addition of PN. In addition, when we used crude GAD-V in a preparative-scale reaction without additional PLP, the concentration of GABA produced was 373 g L^-1^. This provides the basis for the industrial-scale production of GABA. To the best of our knowledge, this is the first report to show that PN plays an important role in enhancing the specific activity, thermostability and application of GAD.

## Supporting Information

S1 Data The initial data of Fig 1. Data A in S1 Data. The initial data about the effects of PN on the activity of GAD. Data B in S1 Data. The initial data of the effects of different concentration of pyridoxine hydrochloride on GAD production.
(XLSX)

S2 Data The data of Fig 3. The initial data of Gel filtration chromatogram of enzyme.
(XLSX)

S3 Data The data of Fig 5. The initial data of CD spectra of GAD.
(XLSX)

S4 Data The initial data of Fig 6. Data A in S4 Data. The initial data of pH optimum of GAD. Data B in S4 Data. The initial data of pH stability of GAD. Data C in S4 Data. The initial data of optimum temperature of GAD. Data D in S4 Data. The initial data of optimum Thermostability of GAD.
(XLSX)

S5 Data The initial data of Fig 7. Data A in S5 Data. The initial data of effect of pH on GABA production. Data B in S5 Data. The initial data of effect of temperature on GABA production. Data C in S5 Data. The initial data of effect of Effect of enzyme dosage on GABA production. Data D in S5 Data. The initial data of effect of substrate concentration on GABA production.
(XLSX)

S6 Data The data of Fig 8. The initial data about GABA synthesis with crude enzyme GAD.
(XLSX)
